# Exogenous heat shock proteins HSPA1A and HSPB1 regulate TNF‐α, IL‐1β and IL‐10 secretion from monocytic cells

**DOI:** 10.1002/2211-5463.13695

**Published:** 2023-08-25

**Authors:** Emmanuel Ogbodo, Francesco Michelangeli, John H. H. Williams

**Affiliations:** ^1^ Coventry University UK; ^2^ Chester Medical School University of Chester UK; ^3^ Chester Centre for Stress Research UK

**Keywords:** cytokine, differentiated U937 cells, heat shock protein, immune response, Naïve U937 cells, PBMCs

## Abstract

Endogenous molecules, such as heat shock proteins (HSP), can function as danger signals when released into the extracellular environment in response to cell stress, where they elicit an immune response such as cytokine secretion. There has also been some suggestion that contamination of exogenous HSPs with lipopolysaccharide (LPS) may be responsible for these effects. This study investigates the effects of exogenous HSPA1A and HSPB1 on the activation of immune cells and the resulting secretion of cytokines, which are involved in inflammatory responses. To address whether exogenous HSPs can directly activate cytokine secretion, naïve U937 cells, differentiated U937 cells and peripheral blood mononuclear cells (PBMCs) were treated with either exogenously applied HSPA1A or HSPB1 and then secreted IL‐1β, TNF‐α and IL‐10 were measured by ELISA. Both HSPs were able to induce a dose‐dependent increase in IL‐10 secretion from naïve U937 cells and dose‐dependent IL‐1β, TNF‐α and IL‐10 secretion were also observed in differentiated U937 cells and PBMCs. We also observed that CD14 affects the secretion levels of IL‐1β, TNF‐α and IL‐10 from cells in response to exogenous HSP treatment. In addition, HSPA1A and HSPB1 were shown to interact with CD14, CD36 and CD11b extracellular receptor proteins. Several approaches used in this study indicate that HSP‐induced cytokine secretion is largely independent of any contaminating LPS in the samples.

## Graphical abstract

Cell stress activates the release of endogenous molecules such as heat shock proteins (HSPs) into the extracellular environment. Here, we investigated cytokine release in naïve, differentiated U937 cells and peripheral blood mononuclear cells following exposure to HSPs. Both HSPA1A and HSPB1 activated cytokine secretion, and data suggests CD14, CD36 and CD11b may play a major role in signalling.

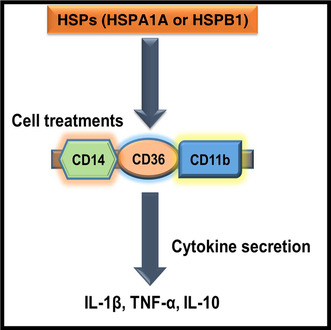

**Abbreviations**
CRISPR: clustered regularly interspaced short palindromic
DAMPs: damage‐associated molecular patterns
dHSP: denatured heat shock protein
ELISA: enzyme‐linked immunosorbent assay
FBS: fetal bovine serum
HIRPMI: heat‐inactivated Roswell Park Memorial Institute
HMGB1: high mobility group box 1
HSPs: heat shock proteins
IL‐10: interleukin 10
IL‐1β: interleukin 1 beta
LPS: lipopolysaccharide
LTA: lipoteichoic acid
MAPK: mitogen‐activated protein kinase
NF‐kB: nuclear factor kappa‐light‐chain‐enhancer of activated B cells
OD: optical density
PAMPs: pathogen‐associated molecular patterns
PBMCs: peripheral blood mononuclear cells
PBS: phosphate‐buffered saline
PMA: phorbol 12‐myristate 13‐acetate
RPMI: Roswell Park Memorial Institute
SD: standard deviation
siRNA: small interfering RNA
SPR: surface plasma resonance
SR‐A1: Saunders‐Roe
TLR: toll‐like receptor
TNF‐α: tumour necrosis factor‐alpha

Heat shock proteins (HSPs) are a group of intracellular proteins that are involved in protein folding and re‐folding, maintaining protein integrity during proteotoxic stress and processing damaged proteins to the proteasome [1]. Not surprisingly, HSPs have been found to have an involvement in several disease systems including cancer, cardiovascular disease, type 1 and type 2 diabetes and infectious disease [1, 2, 3, 4]. Heat shock proteins were also found to be present as extracellular proteins [5, 6], and concentrations of these have been found to vary in many conditions, including HSPB1 and HSPA1A in cancer [7, 8, 9], cardiovascular disease [10], HSPB1 in atherosclerosis [11] and HSPA1A in rheumatoid arthritis [12].

Initially, it was thought that HSPs were only released from necrotic cells following the loss of cell membrane integrity [13, 14], which was a view consistent with the danger model [15]. However, it has been demonstrated that HSPs are secreted from live and apoptotic cells both as a free protein [6, 16, 17, 18, 19, 20] and in exosomes [21, 22]. These extracellular HSPs can be taken up by other cells, where they provide protection, for example HSPA1A in brain cells and monocytes [23]. However, a considerable amount of attention has been paid to possible immune signalling by HSPs and their potential to be acting as danger signals or alarmins.

The immune system response to infection depends on danger signals, which help activate immune cells [24]. Exogenous danger signals include lipopolysaccharide (LPS) and Lipoteichoic acid (LTA), which are typically bacterial antigens or products [25]. Endogenous danger signals, also termed alarmins, include HSPs such as HSPA1A and HSPB1 [26]. Danger signals, including HSP, have been reported to activate inflammatory immune responses via interaction with extracellular receptors such as CD14 and CD91 [27].

Extracellular HSPA1A has been shown to stimulate the secretion of pro‐inflammatory cytokines [28, 29, 30], but has also been shown to have anti‐inflammatory properties [31]. Extracellular HSPB1 has also been shown to have pro‐inflammatory activity [8] and stimulates the release of both pro‐ and anti‐inflammatory cytokines in macrophages [32]. There is, therefore, some conflicting evidence for the roles of extracellular HSPA1A and HSPB1 in immune responses, part of which may be due to the status of the cell. We have used both HSP proteins to investigate cytokine release in naïve, activated U937 monocytic cells and PBMCs.

## Materials and methods

### Cell cultures

Human Caucasian Histiocytic Lymphoma, U937 cell line was purchased from the European Collection of Cell Cultures (Salisbury, UK; 85011440). Naïve U937 human monocytic cells, U937 macrophages (differentiated U937 cells) and human peripheral blood mononucleated cells (PBMCs) were maintained in RPMI 1640 supplemented with 10% fetal bovine serum (FBS) at 37 °C in a humidified atmosphere of 5% CO_2_. Cells were regularly tested for viability using the trypan blue exclusion method.

### U937 cell differentiation

Naïve U937 cells, actively growing in the log phase and with > 95% cell viability were centrifuged at 500 ***g*** for 5 min at 25 °C and the supernatant was discarded. Cells were then re‐suspended in RPMI 1640 with 10% heat‐inactivated FBS (10% HI‐RPMI). Cells were washed three times in HI‐RPMI and then re‐suspended in HI‐RPMI before treatment with 10 ng·mL^−1^ of phorbol 12‐myristate 13‐acetate (PMA; Sigma‐Aldrich Merck, Gillingham, UK) for 24 h. Cell differentiation was monitored through morphological changes using light microscope. The cells were adherent, clusters and appeared larger in size.

### PBMCs isolation from a whole blood

Following the written informed consent obtained from each of the participant donors, blood samples were obtained from healthy volunteers at Chester Medical School after approval by the Faculty Research Ethics Committee (FREC reference: 1382/18/ECO/CMS). PBMCs isolated from the whole blood collected from the University of Chester student's participant blood donors were separated from 20 mL of fresh blood using Histopaque‐1077 (Sigma‐Aldrich Merck). PBMCs were washed three times with phosphate‐buffered saline (PBS) and then re‐suspended in %10 HI‐RPMI followed by cell counting using trypan blue exclusion. The experiments with human PBMC samples conformed to the standards set by the Declaration of Helsinki.

### Preparation of cells for treatment

Cells cultured in 10% FBS and RPMI 1640 were transferred into a centrifuge tube and centrifuged at 500 ***g*** for 5 min at 25 °C. The supernatant was removed and discarded. The cell pellet was gently re‐suspended in 10% HI‐RPMI at a density of 5 × 10^5^ cells·mL^−1^ for U937 cells or 1 × 10^6^ cells·mL^−1^ for PBMCs and washed three times, before resuspending the cell pellet in 10% HI‐RPMI ready for experimental treatment.

Cell viability was determined using trypan blue exclusion dye. The cell counting was performed using a haematocytometer and viewed under a light microscope. Cells that were stained and turned blue were defined as dead cells. The percentage of viable cells was calculated by dividing the viable cells with the total number of cells and then multiplied by 100. There were > 90% viable cells in both naïve and differentiated U937 cells upon either HSPA1A or HSPB1 incubation and after HSPA1A or HSPB1 incubation.

### Preparation of HSP solution for experimental use

Commercially prepared recombinant HSPA1A and HSPB1 (Stressmarq Biosciences, Victoria, Canada) were diluted in 10% HI‐RPMI and added to cells at the final concentrations of 0, 1, 10, 100 and 1000 ng·mL^−1^. For denatured HSPs (dHSPs—e.g. dHSPA1A and dHSPB1) that aimed at excluding possible contamination, the stock solution was heated by boiling in a water bath at 100 °C for 10 min and allowed to cool before adding them to the cells.

### Treatment of differentiated U937 cells with Anti‐HSP

Commercially prepared 1000 ng·mL^−1^ of either human recombinant HSPA1A or HSPB1 (Stressmarq Biosciences) were preincubated with 20 μg·mL^−1^ of their antibodies (i.e. human anti‐HSPA1A or anti‐HSPB1; Stressmarq Biosciences) for 60 min, before then introduced to the differentiated U937 cells for 6 h.

### The treatment of blocking of differentiated U937 cells with blocking peptide

CD14 blocking peptide (Stratech, Cambridge, UK), CD36 blocking peptide (Abcam, Cambridge, UK) and CD11b blocking peptide (Novus Biologicals, Cambridge, UK) were diluted in 10% HI‐RPMI and preincubated with either differentiated U937 cells or HSPA1A or HSPB1 at the final concentration of 20 μg·mL^−1^. Monocytic cells and macrophages express several extracellular receptors. Both exogenous and endogenous molecules such as HSPs can induce cytokine secretion following their interaction with these extracellular receptors [27, 33, 34, 35, 36, 37]. Blocking peptides are used in this study because they competitively block protein–protein interaction, by mimicking protein‐specific binding sites [38]. Therefore, blocking peptides can bind specific receptors following incubation with cells. This can limit molecular interaction with the extracellular receptors and further indicate molecular specificity to the extracellular receptors [39].

### Supernatant collection and cytokine ELISA

Following cell treatment with HSPA1A and HSPB1, the cell suspension was transferred from the tissue culture wells into a microcentrifuge tube and centrifuged at 500 ***g*** for 5 min. The concentration of IL‐1b,TNF‐α and IL‐10 in the cell culture supernatants was measured by ELISA kit (eBioscience‐Fisher Scientific, Loughborough, UK) following the manufacture's procedures. Optical density (OD) at 450 nm was determined using a Varioskanlux (Thermo‐Scientific, Winsford, UK) microplate plate reader.

### Determination of cell surface receptor proteins expression by flow cytometry

Flow cytometry was used to measure CD14, CD36 and CD11b on differentiated U937 cells. Differentiated U937 cells were either treated or not treated with 1000 ng·mL^−1^ of HSPA1A or HSPB1 and incubated for 6 h. Cells were either immune‐stained with or without 5 μL Anti‐CD14: APC‐CyTM7, Anti‐CD36: PE, Anti‐CD11b: FITC (BD BioSciences, Franklin Lakes, NJ, USA). Cells were also stained with 10 μL propidium iodide staining solution (BD BioSciences) for 1 h at 4 °C in the dark. The cells were gated using flow cytometry BD Accuri™ c6 (Piscataway, NJ, USA) to select by circling viable cells. This ensured only viable cells were analysed and not cell debris.

### Statistical analysis

Data are presented as the mean ± SD, *n* = 3. Each set of the experiments were done at least three times with three replicates undertaken for each of the independent experiments tested. Analysis was performed with graphpad prism™ version 7.0 (GraphPad Software, Inc, San Diego, CA, USA). The two‐way ANOVA was carried out to analyse the significance in the differences between HSP‐treated and control cells (10% HI‐RPMI). The statistical significance level was set as *P < 0.05; **P < 0.01; and ***P < 0.001.

## Results

### Exogenous HSPA1A and HSPB1 concentration on IL‐1β, TNF‐α and IL‐10 secretion

**Fig. 1:**
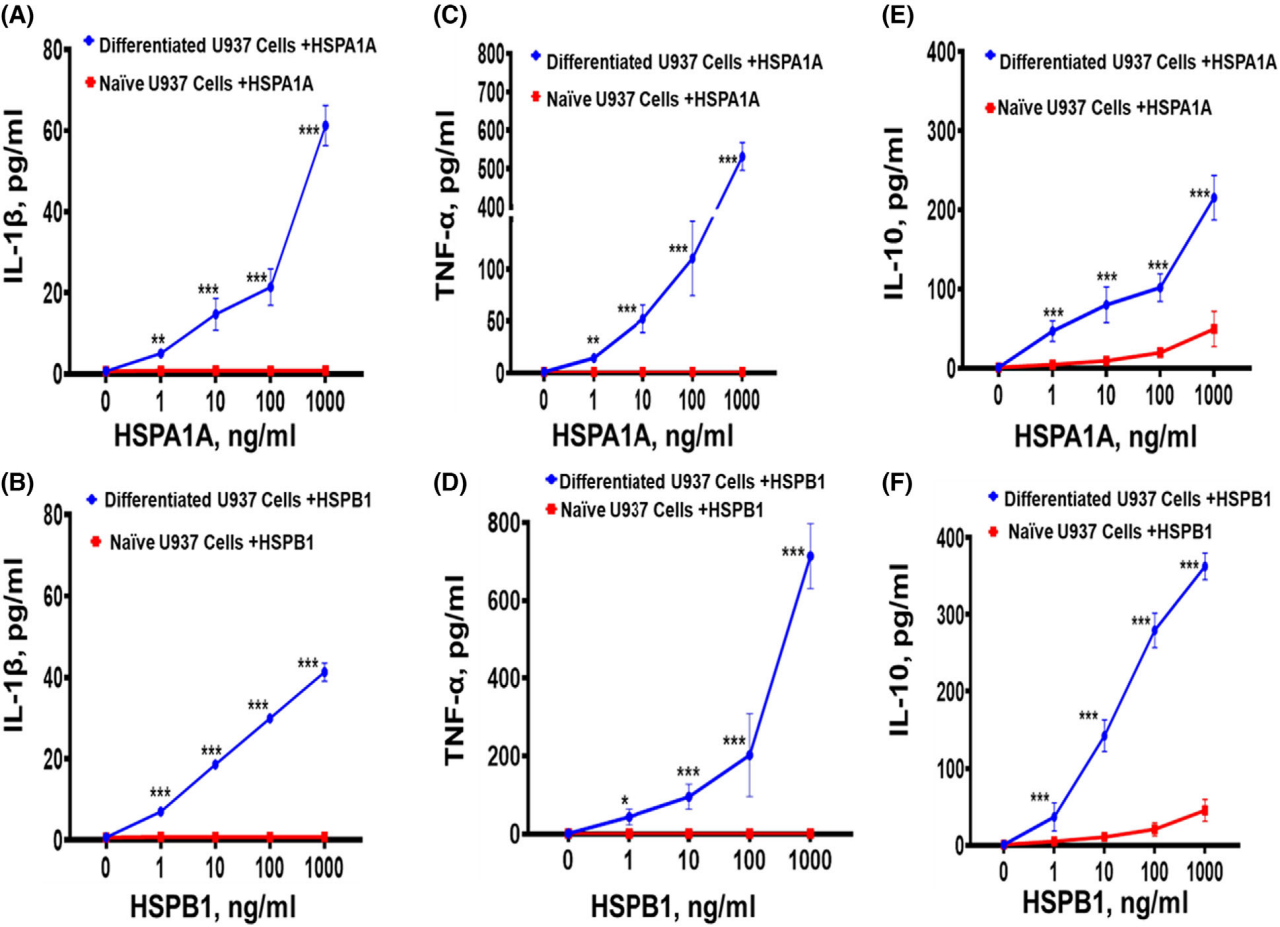
Effects of HSPA1A and HSPB1 concentration on the secretion of IL‐1β, TNF‐α and IL‐10 from undifferentiated (naïve) U937 cells and differentiated U937 cells (U937 macrophages) following treatment of different concentrations of either HSPA1A or HSPB1 for 24 h. Panel (A) shows the treatment of HSPA1A concentration effect in IL‐1β secretion. Panel (B) shows the treatment of HSPB1 concentration effect in IL‐1β secretion. Panel (C) shows the treatment of HSPA1A concentration effect in TNF‐α secretion. Panel (D) shows the treatment of HSPB1 concentration effect in TNF‐α secretion. Panel (E) shows the treatment of HSPA1A concentration effect in IL‐10 secretion. Panel (F) shows the treatment of HSPB1 concentration effect in IL‐10 secretion. Data are presented as mean ± SD, *n* = 3, and tested by one‐way ANOVA with Bonferroni's multiple comparison *post hoc*. Significant differences between HSPA1A and HSPB1 concentrations respectively on differentiated and Naïve U937 cells treatment are shown *P < 0.5, **P < 0.01 and ***P < 0.001.

Initially, an investigation of HSPA1A and HSPB1‐induced cytokine secretion from naïve U937 cells was undertaken, which, unlike other studies, had used U937 cells which had first been differentiated into macrophages [28, 40, 41]. The results of this study showed that HSPA1A and HSPB1 concentrations were not able to induce IL‐1β and TNF‐α secretion from naïve U937 cells (Fig. 1A–D). They were, however, able to induce IL‐10 secretion (*P* < 0.001) in a dose‐dependent manner with increasing HSPA1A or HSPB1 concentrations (Fig. 1E,F), compared with U937 cells not treated with either HSPA1A or HSPB1. The same treatments with HSPA1A and HSPB1 were clearly able to induce IL‐1β, TNF‐α and IL‐10 secretion (*P* < 0.001) from differentiated U937 cells in a dose‐dependent manner, with increasing HSPA1A and HSPB1 concentration (Fig. 1A–F). There was more than a 5‐ to 10‐fold increase in IL‐10 secretion from differentiated U937 cells treated with either HSPA1A or HSPB1 than in naïve U937 cells treated with either HSPA1A or HSPB1.

In order to assess whether the differences in the magnitudes of the cytokine secretion in both naïve and differentiated U937 cells were due to temporal differences in cytokine expression and/or release into the extracellular medium, a time course study was undertaken, where the cells were treated with either 1000 ng·mL^−1^ of HSPA1A and HSPB1 and cytokine secretion followed over 72 h.

### Time course effect of exogenous HSPA1A and HSPB1‐induced IL‐1β, TNF‐α and IL‐10 secretion from U937 cells and differentiated U937 macrophages

**Fig. 2:**
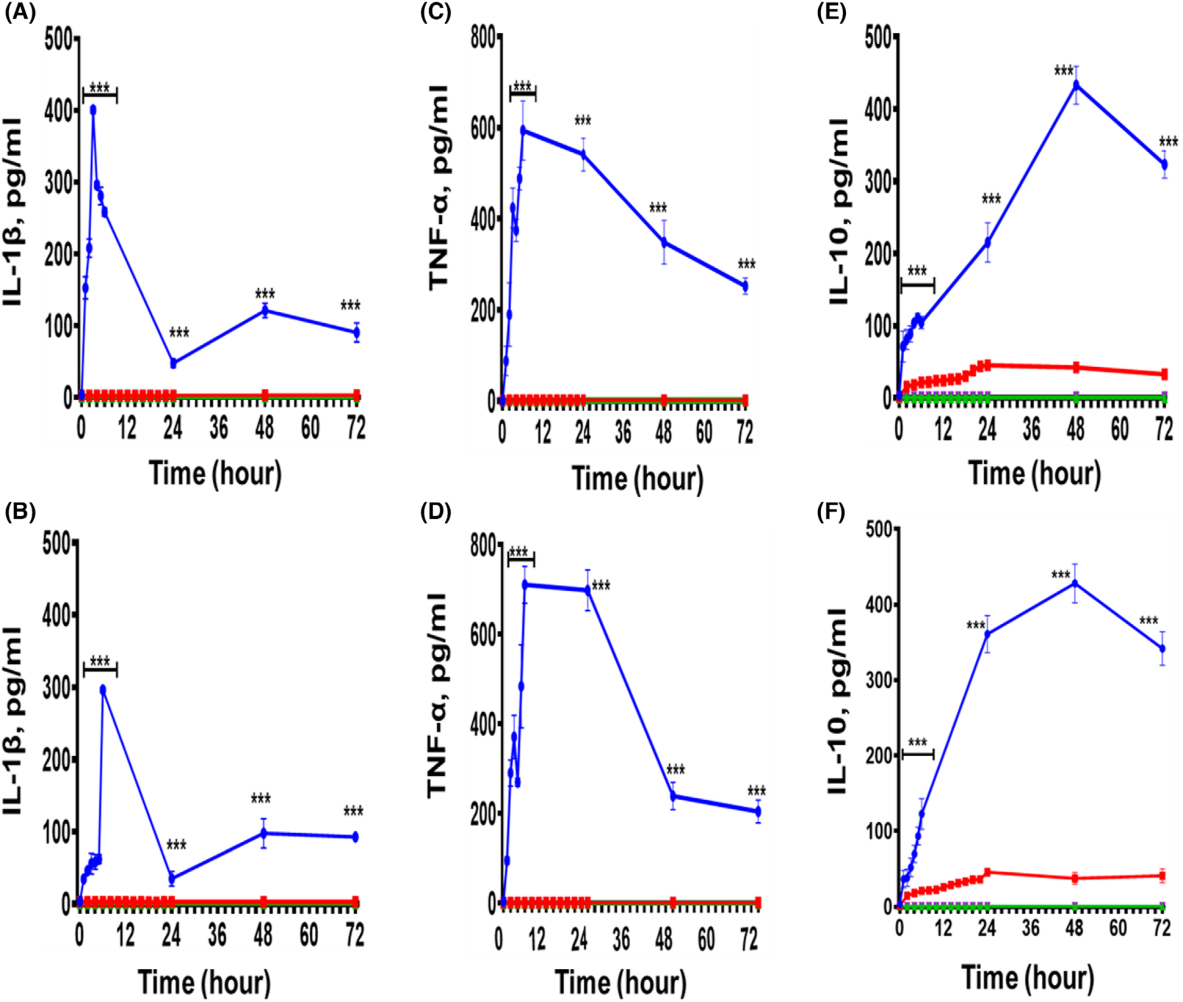
Time course effects of 1000 ng·mL^−1^ HSPA1A and 1000 ng·mL^−1^ HSPB1 induced IL‐1β, TNF‐α and IL‐10 secretion from undifferentiated (naïve) U937 cells and differentiated U937 cells (U937 macrophages) following treatment for 72 h. Panels (A, C and E) are results gained in the treatment of naïve U937 cells and differentiated U937 cells with 1000 ng·mL^−1^ of HSPA1A. Panels (B, D and F) are results gained in the treatment of naïve U937 cells and differentiated U937 cells with 1000 ng·mL^−1^ of HSPB1. In addition, Panels (A and B) present the time course effect of either HSPA1A or HSPB1 in the activation of IL‐1β secretion into the media. Panels (C and D) present the time course effect of either HSPA1A or HSPB1 in the activation of TNF‐α secretion into the media. Panels (E and F) present the time course effect of either HSPA1A or HSPB1 in the activation of IL‐10 secretion into the media. Panels (A) show the effects of HSPA1A in IL‐1β, (C) the effects of HSPA1A in TNF‐α and (E) the effects of HSPA1A in IL‐10, while panel (B) show the effects of HSPB1 in IL‐1β, (D) the effects of HSPB1 in TNF‐α and (F) the effects of HSPB1 in IL‐10. Data are presented as mean ± SD, *n* = 3 and tested by two‐way ANOVA with Bonferroni's multiple comparison *post hoc*. Significant differences between HSPA1A and HSPB1 respectively and control (HI‐RPMI) treatment are shown ***P < 0.001.

The treatment of naïve U937 cells with 1000 ng·mL^−1^ of either HSPA1A or HSPB1 resulted in an increase IL‐10 secretion (*P* < 0.001) at different time points up to 72 h, when compared to U937 cells treated with 10% HI‐RPMI alone (Fig. 2E,F). The rate of initial IL‐10 secretion in naïve U937 cells treated with either HSPA1A or HSPB1 was 1.87 pg·mL^−1^·h^−1^ in HSPA1A and 1.90 pg·mL^−1^·h^−1^ in HSPB1 treatments. The same treatment had no effect on IL‐1β and TNF‐α secretion (Fig. 2A–D). The results showed that at shorter times, there was increased IL‐10 secretion within 2 h, after which IL‐10 secretion increased more gradually (Fig. 2E,F). Significance differences were observed at 2 h until 72 h between either HSPA1A or HSPB1, and HI‐RPMI (*P* < 0.001; Fig. 2E,F). The IL‐10 secretion in naïve U937 cells remained relatively constant after 24 h (Fig. 2E,F).

The effect of differentiated U937 cells (U937 macrophages) treatment with 1000 ng·mL^−1^ of either HSPA1A or HSPB1 showed increased IL‐1β and TNF‐α secretion (*P* < 0.001) at different time points up to 72 h, when compared to differentiated U937 cells treated with 10% HI‐RPMI only (Fig. 2A–D). The initial rate of IL‐1β secretion in differentiated U937 cells treated with HSPA1A or HSPB1 was 135.0 pg·mL^−1^·h^−1^ in HSPA1A (Fig. 2A) and 48.8 pg·mL^−1^·h^−1^ in HSPB1 (Fig. 2B). The initial rate of TNF‐α secretion in differentiated U937 cells treated with either HSPA1A or HSPB1 were 132.6 pg·mL^−1^·h^−1^ in HSPA1A (Fig. 2C) and 123.3 pg·mL^−1^·h^−1^ in HSPB1 (Fig. 2D) treatments. The same treatments in differentiated U937 cells also showed an increase in IL‐10 secretion (*P* < 0.001; Fig. 2E,F), and the initial rate of IL‐10 secretion was 22.0 pg·mL^−1^·h^−1^ in HSPA1A (Fig. 2E) and 15.3 pg·mL^−1^·h^−1^ in HSPB1 (Fig. 2F) treatments. The results showed that there was IL‐1β, TNF‐α and IL‐10 secretion at shorter times within 1 h until 72 h. Also, over longer time periods, the level of cytokines appeared to decrease (Fig. 2A–F).

The results presented in Fig. 2 showed that the initial rate of IL‐10 secretion in differentiated U937 cells treated with HSPA1A was 20 times more than that of the naïve U937 cells treated with HSPA1A (Fig. 2E) and 13 times more than in differentiated U937 cells treated with HSPA1A, compared with naïve U937 cells treated with HSPB1 (Fig. 2F). In addition, time was demonstrated to contribute to the amount of cytokine secreted from both U937 cells and U937 macrophages. Therefore, to further investigate whether this was also observed in primary cells, additional studies were undertaken using PBMC cells to help understand whether the effect of extracellular HSPA1A and HSPB1 is reflective of those seen in primary cells.

### Time course effect of exogenous HSPA1A and HSPB1‐induced IL‐1β, TNF‐α and IL‐10 secretion from PBMCs

**Fig. 3:**
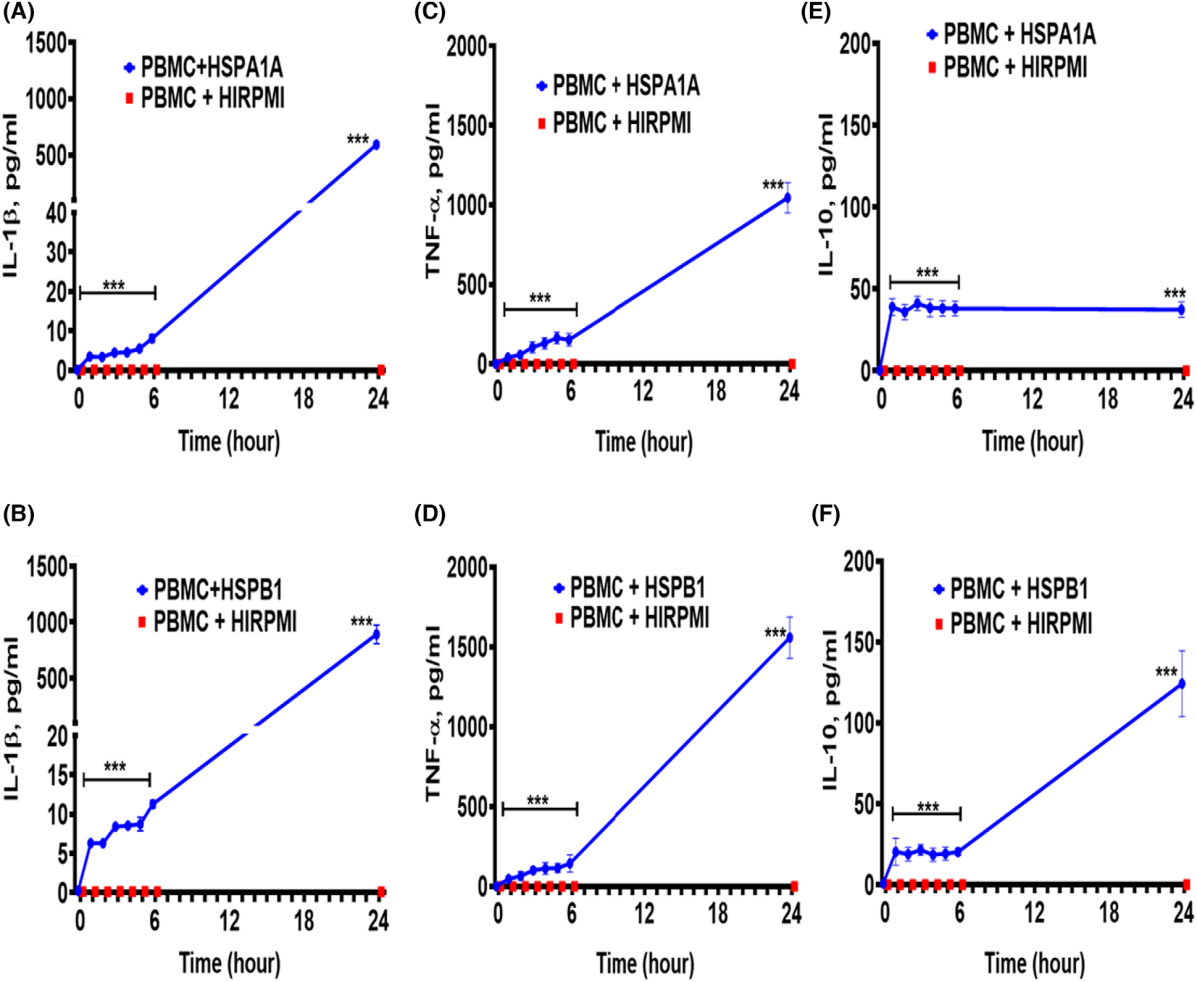
Time course effects of 1000 ng·mL^−1^ HSPA1A and HSPB1 induced IL‐1β, TNF‐α and IL‐10 secretion from PBMCs following treatment for 24 h. Panels (A, C and E) present the time course effect of HSPA1A in the activation of IL‐1β (panel A), TNF‐α (panel C) and IL‐10 (panel E) secretion into the media. Panels (B, D and F) present the time course effect of HSPB1 in the activation of IL‐1β (panel B), TNF‐α (panel D) and IL‐10 (panel F) secretion into the media. Data are presented as mean ± SD, *n* = 3, and tested by two‐way ANOVA with Bonferroni's multiple comparison *post hoc*. Significant differences between HSPA1A and HSPB1 respectively and control (HI‐RPMI) treatment are shown ***P < 0.001.

**Fig. 4:**
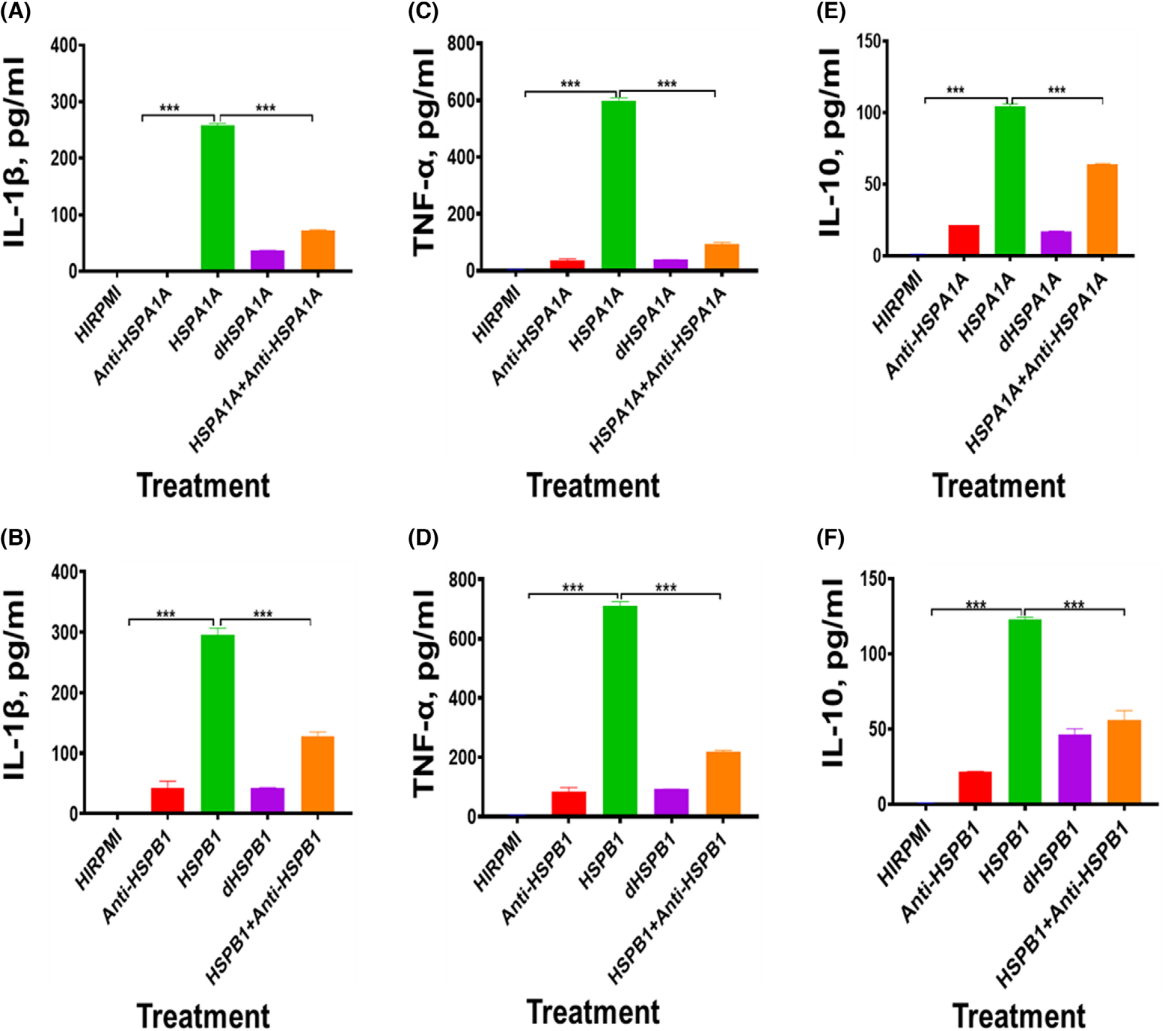
Effects of Anti‐HSP, HSP + Anti‐HSP, dHSP and HIRPMI on 1000 ng·mL^−1^ of either HSPA1A or HSPB1 Induced IL‐1β, TNF‐α and IL‐10 secretion from differentiated U937 cells (U937 macrophages) at 24 h. Panels (A, C and E) are the results gained in the treatment of differentiated U937 cells with HSPA1A, (HSPA1A + Anti‐HSPA1A), dHSPA1A, Anti‐HSPA1A and HIRPMI. Panels (B, D and F) are the results gained in the treatment of differentiated U937 cells with HSPB1, (HSPB1 + Anti‐HSPB1), dHSPB1, Anti‐HSPB1 and HIRPMI. Panels (A) showed the effects of cell treatments with HSPA1A, (HSPA1A + Anti‐HSPA1A), dHSPA1A, Anti‐HSPA1A and HIRPMI in IL‐1β secretion, Panel (C) showed the effects of cell treatments with HSPA1A, (HSPA1A + Anti‐HSPA1A), dHSPA1A, Anti‐HSPA1A and HIRPMI in TNF‐α secretion, and Panel (E) showed the effects of cell treatments with HSPA1A, (HSPA1A + Anti‐HSPA1A), dHSPA1A, Anti‐HSPA1A and HIRPMI in IL‐10 secretion. While Panel (B) showed the effects of cell treatments with HSPB1, (HSPB1 + Anti‐HSPB1), dHSPB1, Anti‐HSPB1 and HIRPMI in IL‐1β secretion, Panel (D) showed the effects of cell treatments with HSPB1, (HSPB1 + Anti‐HSPB1), dHSPB1, Anti‐HSPB1 and HIRPMI in TNF‐α secretion, and Panel (F) showed the effects of cell treatments with HSPB1, (HSPB1 + Anti‐HSPB1), dHSPB1, Anti‐HSPB1 and HIRPMI in IL‐10 secretion. Data are presented as mean ± SD, *n* = 3, and tested by one‐way ANOVA with Bonferroni's multiple comparison *post hoc*. Significant differences between HSP and (HSP + Anti‐HSP), dHSP, Anti‐HSP and control (HIRPMI) respectively are shown ***P < 0.001.

The treatment of PBMCs with 1000 ng·mL^−1^ of either HSPA1A or HSPB1 showed IL‐1β and TNF‐α secretion (*P* < 0.001) at a different time up to 24 h, when compared to PBMCs treated with 10% HI‐RPMI only (Fig. 3A–D). The initial rate of IL‐1β secretion in PBMCs treated with HSPA1A or HSPB1 was 1.16 pg·mL^−1^·h^−1^ in HSPA1A (Fig. 3A) and 2.13 pg·mL^−1^·h^−1^ in HSPB1 (Fig. 3B). The initial rate of TNF‐α secretion in PBMCs treated with either HSPA1A or HSPB1 was 32.7 pg·mL^−1^·h^−1^ in HSPA1A (Fig. 3C) and 24.0 pg·mL^−1^·h^−1^ in HSPB1 (Fig. 3D) treatments. The same treatment also showed an increase in IL‐10 secretion (*P* < 0.001; Fig. 3E,F) and the initial rate of IL‐10 secretion in PBMCs treated with either HSPA1A or HSPA1A was 38.9 pg·mL^−1^·h^−1^ in HSPA1A (Fig. 3E) and 19.4 pg·mL^−1^·h^−1^ in HSPB1 (Fig. 3F) treatments. The results also showed that there was IL‐1β, TNF‐α and IL‐10 secretion within 1 until 24 h. In addition, HSPA1A‐induced IL‐10 secretion appeared very rapid within 1 h and then constant (Fig. 3E) but was very slow for HSPB1‐induced IL‐10 secretion only after 24 h (Fig. 4F). IL‐1β and TNF‐α, however, showed a more gradual increase with both HSPA1A and HSPB1(Fig. 3A–D) and PBMCs treatment with either HSPA1A or HSPB1 had more than double effects on IL‐1β and TNF‐α secretion (Fig. 3A–D) compared with the treatments of differentiated U937 cells with either HSPA1A or HSPB1 (Fig. 2A–D).

### The effects of Anti‐HSP and denatured HSP on HSP‐induced IL‐1β, TNF‐α and IL‐10 secretion

In these experiments, the effect of anti‐HSP antibodies on the HSP‐induced cytokine secretion was employed by preincubating either HSPA1A or HSPB1 with their specific antibodies. IL‐1β, TNF‐α and IL‐10 were then measured by ELISA. This approach was because proteins are specific to their antibodies. Secondly, both HSPA1A and HSPB1 were first denatured by boiling at 100 °C because one of the main characteristics of LPS is their heat resistance. This can help highlight if HSP‐induced cytokine secretion is mainly due to LPS contamination, since HSPs are much more heat labile and become denatured under these conditions.

The treatment of differentiated U937 cells with 1000 ng·mL^−1^ of either HSPA1A or HSPB1 resulted in increased (*P* < 0.001) IL‐1β, TNF‐α and IL‐10 secretion compared with either dHSPA1A or dHSPB1 respectively at 24 h (Fig. 4A–F). The IL‐1β secretion in differentiated U937 cells treated with either HSPA1A or dHSPA1A and HSPB1 or dHSPB1 was 258.30 pg·mL^−1^·h^−1^ in HSPA1A; 37.01 pg·mL^−1^·h^−1^ in dHSPA1A (Fig. 4A); 296.10 pg·mL^−1^·h^−1^ in HSPB1; and 42.43 pg·mL^−1^·h^−1^ in dHSPB1 (Fig. 4B) treatments. The TNF‐α secretion in differentiated U937 cells treated with either HSPA1A or dHSPA1A and HSPB1 or dHSPB1 was 596.70 pg·mL^−1^·h^−1^ in HSPA1A; 38.19 pg·mL^−1^·h^−1^ in dHSPA1A (Fig. 4C) and 710.60 pg·mL^−1^·h^−1^ in HSPB1; 91.16 pg·mL^−1^·h^−1^ in dHSPB1 (Fig. 4D) treatments. The same treatments showed IL‐10 secretion of 104.40 pg·mL^−1^·h^−1^ in HSPA1A; 16.65 pg·mL^−1^·h^−1^ in dHSPA1A; 122.80 pg·mL^−1^·h^−1^ in HSPB1; and 46.72 pg·mL^−1^ in dHSPB1 (Fig. 4E,F).

To further determine whether HSP‐induced cytokine is due to HSP or due to LPS contamination, HSPA1A and HSPB1‐induced IL‐1β, TNF‐α and IL‐10 secretion was determined following preincubation of either HSPA1A or HSPB1 with 20 μg·mL^−1^ of their human‐specific antibodies. This would also help to give confidence if HSP can induce cytokine secretion independent of LPS (Fig. 4).

The preincubation of either HSPA1A or HSPB1 with 20 μg·mL^−1^ of their specific anti‐HSP antibodies before then applied to differentiated U937 cells showed a decrease in IL‐1β secretion (*P* < 0.001), when compared to cells treated with either HSPA1A or HSPB1 (Fig. 4A,B). The same treatment showed a decrease in TNF‐α secretion (*P* < 0.001), when compared to cells treated with either HSPA1A or HSPB1 only (Fig. 4C,D). The same treatments also showed a decrease in IL‐10 secretion (*P* < 0.001) following preincubation of either HSPA1A or HSPB1 with anti‐HSPA1A or anti‐HSPB1 respectively, when compared to differentiated U937 cells treated with HSPA1A or HSPB1 only (Fig. 4E,F).

The treatment of differentiated U937 cells with 1000 ng·mL^−1^ of either HSPA1A or HSPB1 resulted in increased (*P* < 0.001) IL‐1β, TNF‐α and IL‐10 secretion compared with either differentiated U937 cells treated with anti‐HSPA1A or anti‐HSPB1 and HIRPMI respectively at 24 h (Fig. 4A–F).

### Expression of cell surface CD14, CD36 and CD11b from differentiated U937 cells

**Fig. 5:**
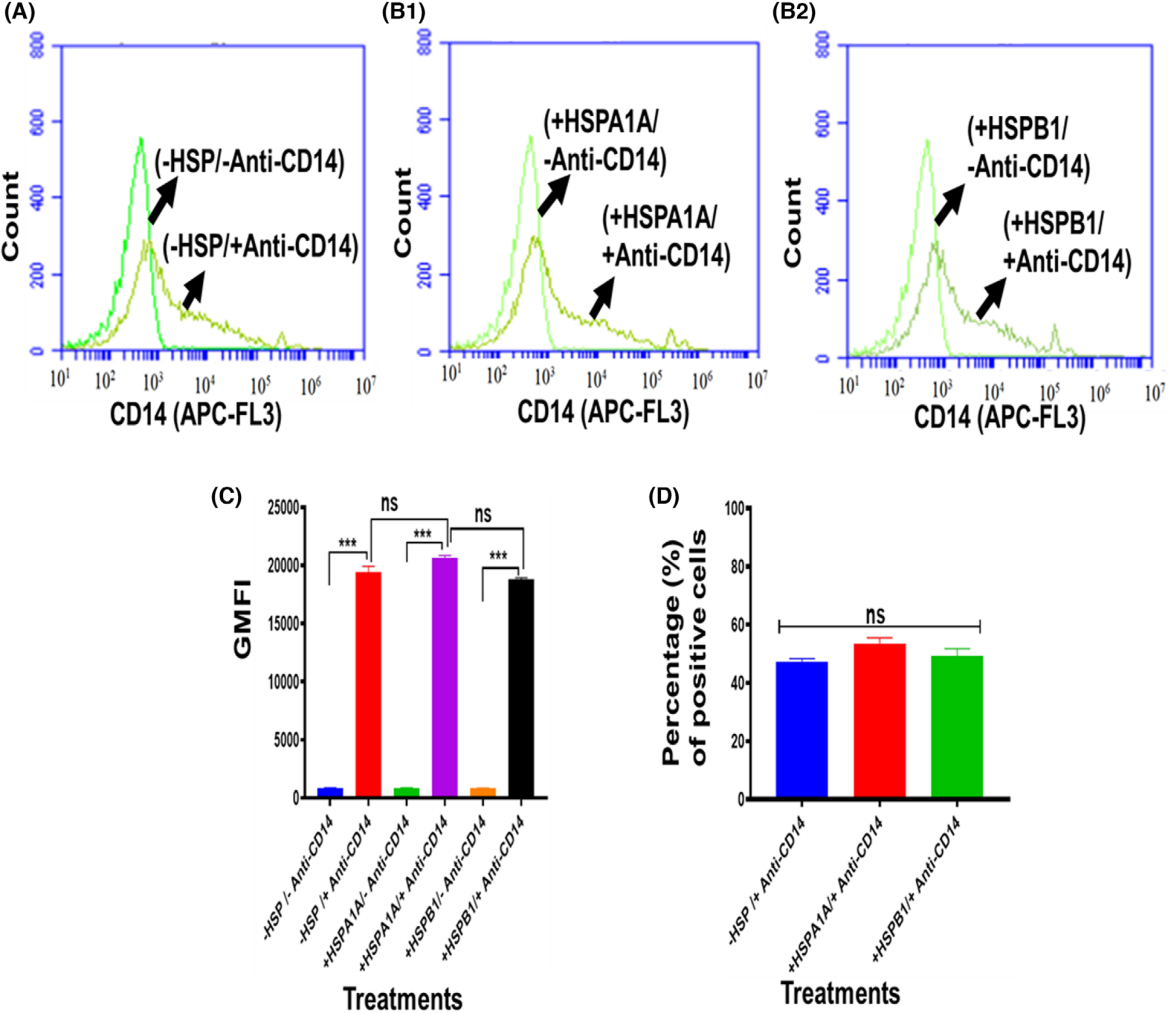
Expression of cell surface CD14 from differentiated U937 cells. 1 × 10^6^·mL^−1^ of differentiated U937 cells were suspended in 10% HI‐RPMI before either treated or not treated with 1000 ng·mL^−1^ HSPA1A or HSPB1 and incubated for 6 h. The cell suspension was then centrifuged, and the supernatant was discarded. The cell pellet was then immuno‐stained with Anti‐CD14 antibody, and flow cytometry analysis was then performed. Panels showed that a proportion of differentiated U937 cells were highly labelled (A) (−HSP/−Anti‐CD14 & −HSP/+Anti‐CD14). (B1) (+HSPA1A/−Anti‐CD14 & +HSPA1A/+Anti‐CD14). (B2) (+HSPB1/−Anti‐CD14 & +HSPB1/+Anti‐CD14). Compared with cells without immuno‐stained with Anti‐CD14 antibody. Panel (C) shows the geometric mean fluorescence intensity (GMFI) derived from the traces in panels (A, B1 and B2) using software from the BD Accuri™ c6 plus analyser. Panel (D) showed the percentage of the CD14+ from differentiated U937 cells treated or not treated with either HSPA1A or HSPB1. Data (Panels A, B and B2) are representative of one experiment. Overall, the experiment was performed and analysed in three replicates for each experiment. Data are presented as mean ± SD, *n* = 3, and statistical analysis was performed by one‐way ANOVA with Bonferroni's multiple comparison *post hoc* test. There was a significant difference (Panel C) between −/+HSP/+Anti‐CD14 and −/+HSP/−Anti‐CD14 are shown ***P < 0.001. However, no significant difference (*P* > 0.05) between −HSP/+CD14 and +HSP/+CD14. Also, the percentage (%) of positive cells showed (Panel D) no significant difference (*P* > 0.05) between −HSP/+Anti‐CD14 and +HSP/+Anti‐CD14 in cells treated with either HSPA1A or HSPB1.

**Fig. 6:**
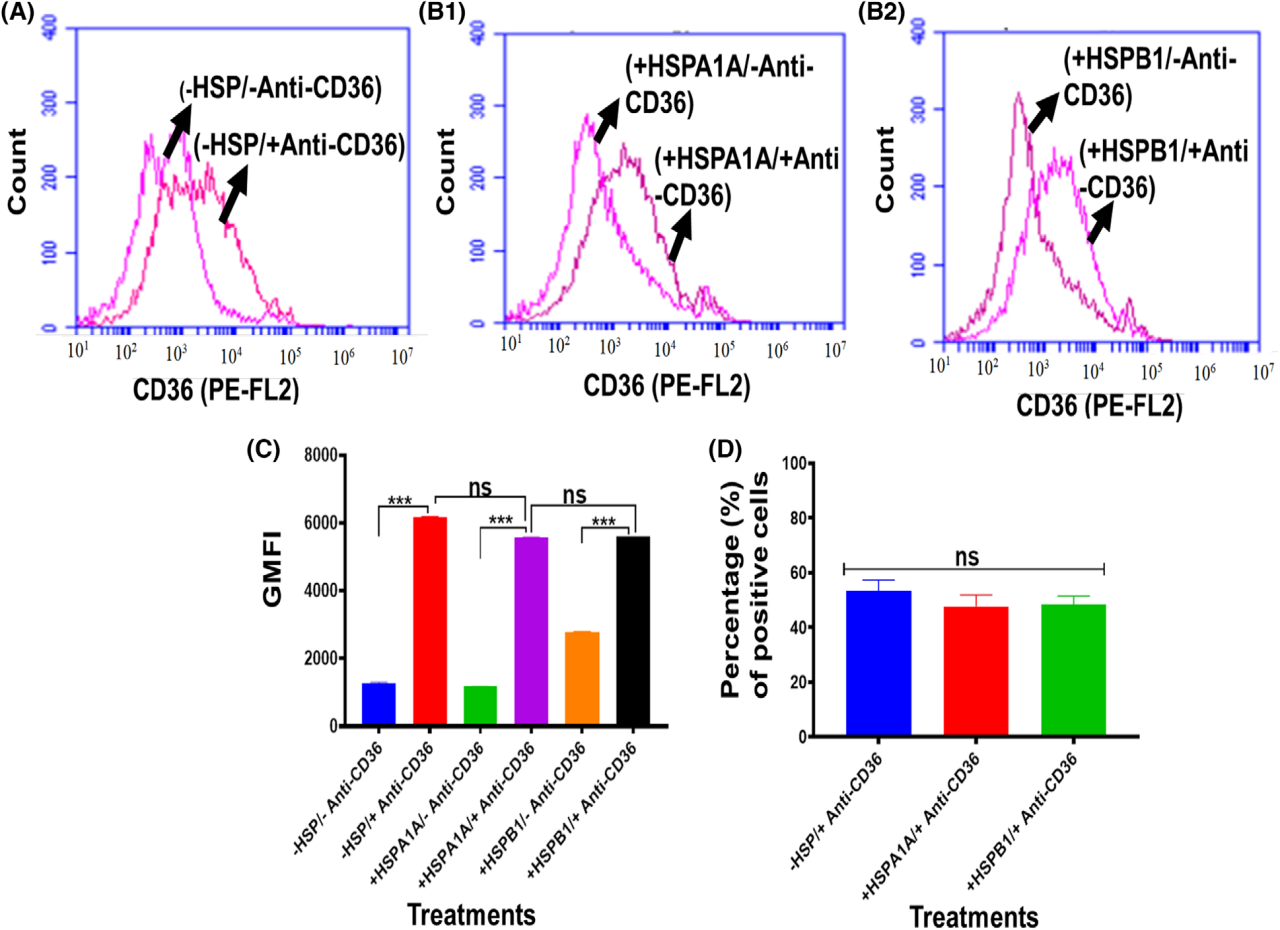
Expression of cell surface CD36 from differentiated U937 cells. 1 × 10^6^·mL^−1^ of differentiated U937 cells were suspended in 10% HI‐RPMI before either treated or not treated with 1000 ng·mL^−1^ HSPA1A or HSPB1 and incubated for 6 h. The cell suspension was then centrifuged, and the supernatant was discarded. The cell pellet was then immuno‐stained with Anti‐CD36 antibody, and flow cytometry analysis was then performed. Panels showed that a proportion of differentiated U937 cells were highly labelled (A) (−HSP/−Anti‐CD36 & −HSP/+Anti‐CD36). (B1) (+HSPA1A/−Anti‐CD36 & +HSPA1A/+Anti‐CD36). (B2) (+HSPB1/−Anti‐CD36 & +HSPB1/+Anti‐CD36). Compared with cells without immuno‐stained with Anti‐CD36 antibody. Panel (C) shows the geometric mean fluorescence intensity (GMFI) derived from the traces in panels (A, B1 and B2) using software from the BD Accuri™ c6 plus analyser. Panel (D) showed the percentage of the CD36^+^ from differentiated U937 cells treated or not treated with either HSPA1A or HSPB1. Data (Panels A, B1 and B2) are representative of one experiment. Overall, the experiment was performed and analysed in three replicates for each experiment. Data are presented as mean ± SD, *n* = 3, and statistical analysis was performed by one‐way ANOVA with Bonferroni's multiple comparison *post hoc* test. There was a significant difference (Panel C) between −/+HSP/+Anti‐CD36 and −/+HSP/−Anti‐CD36 are shown ***P < 0.001. However, no significant difference (*P* > 0.05) between −HSP/+CD36 and +HSP/+CD36. Also, the percentage (%) of positive cells showed (Panel D) no significant difference (*P* > 0.05) between −HSP/+Anti‐CD36 and +HSP/+Anti‐CD36 in cells treated with either HSPA1A or HSPB1.

**Fig. 7:**
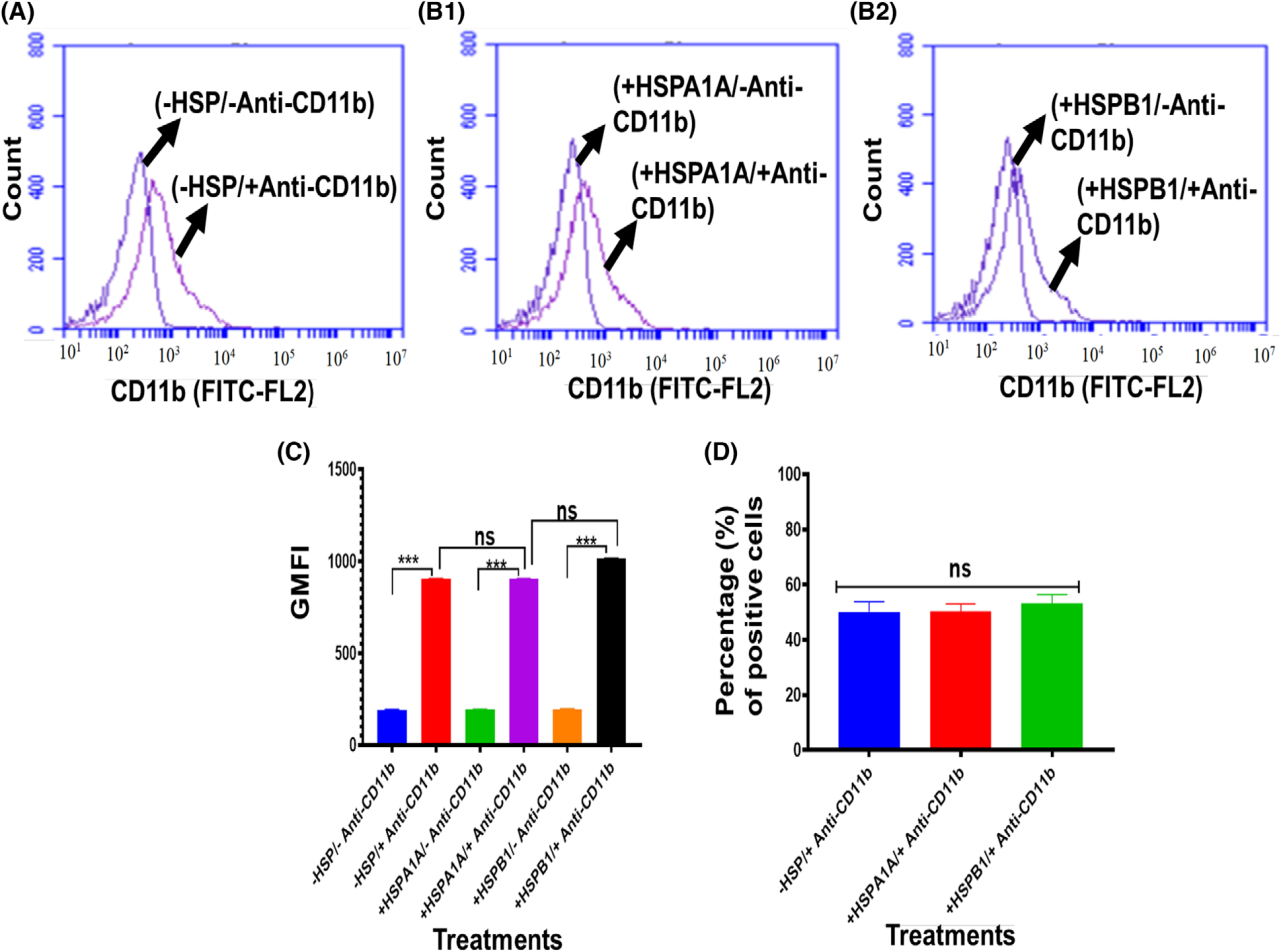
Expression of cell surface CD11b from differentiated U937 cells. 1 × 10^6^·mL^−1^ of differentiated U937 cells were suspended in 10% HI‐RPMI before either treated or not treated with 1000 ng·mL^−1^ HSPA1A or HSPB1 and incubated for 6 h. The cell suspension was then centrifuged, and the supernatant was discarded. The cell pellet was then immuno‐stained with Anti‐CD11b antibody, and flow cytometry analysis was then performed. Panels showed that a proportion of differentiated U937 cells were highly labelled (A) (−HSP/−Anti‐CD11b & −HSP/+Anti‐CD11b). (B1) (+HSPA1A/−Anti‐CD11b & +HSPA1A/+Anti‐CD11b). (B2) (+HSPB1/−Anti‐CD11b & +HSPB1/+Anti‐CD11b). Compared with cells without immuno‐stained with Anti‐CD11b antibody. Panel (C) shows the geometric mean fluorescence intensity (GMFI) derived from the traces in panels (A, B1 and B2) using software from the BD Accuri™ c6 plus analyser. Panel (D) showed the percentage of the CD11b + from differentiated U937 cells treated or not treated with either HSPA1A or HSPB1. Data (Panels A, B1 and B2) are representative of one experiment. Overall, the experiment was performed and analysed in three replicates for each experiment. Data (Panels C and D) are presented as mean ± SD, *n* = 3, and statistical analysis was performed by one‐way ANOVA with Bonferroni's multiple comparison *post hoc* test. There was a significant difference (Panel C) between −/+HSP/+Anti‐CD11b and −/+HSP/−Anti‐CD11b are shown ***P < 0.001. However, no significant difference (*P* > 0.05) between −HSP/+CD11b and +HSP/+CD11b. Also, the percentage (%) of positive cells showed (Panel D) no significant difference (*P* > 0.05) between −HSP/+Anti‐CD11b and +HSP/+Anti‐CD11b in cells treated with either HSPA1A or HSPB1.

The activation of the monocytic cells to secrete pro‐ and anti‐inflammatory cytokines required the interaction of antigens with receptor proteins on the cell surface. The expression of cell surface CD14, CD36 and CD11b was next determined on differentiated U937 cells by flow cytometry. The cells were immuno‐stained with Anti‐CD14 (Fig. 5), Anti‐CD36 (Fig. 6) and Anti‐CD11b (Fig. 7) antibodies and analysed against the cells that were not immuno‐stained with Anti‐CD14 antibody, Anti‐CD36 and Anti‐CD11b. The results showed that the differentiated U937 cell was CD14 positive (Fig. 5A,B1,B2), CD36 positive (Fig. 6A,B1,B2) and CD11b positive (Fig. 7A,B1,B2).

The geometric mean fluorescence intensity (GMFI; Figs 5C, 6C and 7C) derived from these traces (Figs 5A,B1,B2, 6A,B1,B2 and 7A,B1,B2) shows that there was a proportion of differentiated U937 cells that were more highly labelled when compared to differentiated U937 cells without immuno‐stained with the Anti‐CD14, Anti‐CD36 and Anti‐CD11b antibody respectively. This indicates that these Anti‐CD14, Anti‐CD36 and Anti‐CD11b antibodies were able to distinguish the expression of CD14, CD36 and CD11b in differentiated U937 cells compared with the control (*P* < 0.001).

The percentage of the proportion of the cells that were CD14 positive (Fig. 5A,B1,B2), CD36 positive (Fig. 6A,B1,B2) and CD11b positive (Fig. 7A,B1,B2) was assessed (Figs 5D, 6D and 7D). The results showed relatively 50% CD14^+^ (Fig. 5D). Anti‐CD36 immuno‐stained showed relatively 55% of differentiated U937 cells positive (Fig. 6D). The same cells were stained with Anti‐CD11b, and the results showed relatively 51% positive (Fig. 7D).

As differentiated, U937 cells showed expression of CD14, CD36 and CD11b. The next experiment focused on the effects of CD14, CD36 and CD11b blocking peptides on HSPA1A and HSPB1 induced IL‐1β, TNF‐α and IL‐10 secretion in differentiated U937 cells.

### The effect of CD14, CD36 and CD11b blocking peptide on either HSPA1A‐ or HSPB1‐induced cytokine

In these experiments, the effects of inhibiting CD14, CD36 and CD11b on the cell surface with specific blocking peptides, respectively, on either HSPA1A‐ or HSPB1‐induced cytokine secretion in differentiated U937 cells were assessed.

**Fig. 8:**
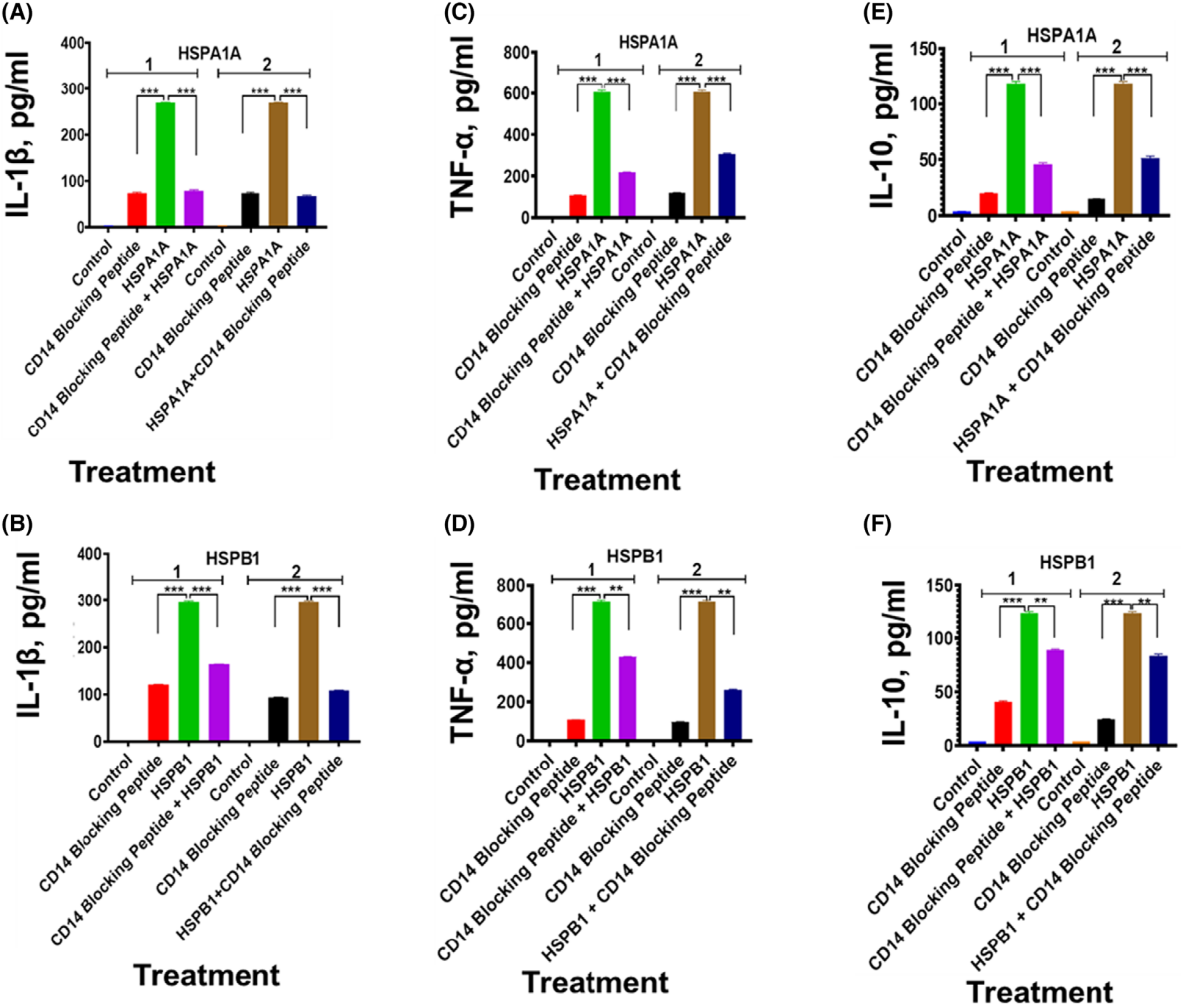
Effect of CD14 blocking peptide on either HSPA1A or HSPB1 induced IL‐1β, TNF‐α and IL‐10 secretion from differentiated U937 cells. Panels (A1–F1) are results gained with differentiated U937 cells treated with 20 μg·mL^−1^ of CD14 blocking peptide alone and then incubated for 2 h followed by the addition of either 1000 ng·mL^−1^ of HSPA1A (A1, C1 and E1) or HSPB1 (B1, D1 and F1) and incubated for further 6 h. The cell suspension was then centrifuged, and the supernatant retrieved was measured for IL‐1β, TNF‐α and IL‐10 secretion into the media by ELISA. In addition, Panels (A2–F2) are results gained with 1000 ng·mL^−1^ of either HSPA1A (A2, C2 and E2) or HSPB1 (B2, D2 and F2) first preincubated with 20 μg·mL^−1^ of CD14 blocking peptide for 2 h, before applied to the differentiated U937 cells and incubated for 6 h. The cell suspension was then centrifuged, and the supernatant retrieved was measured for IL‐1β, TNF‐α and IL‐10 secretion by ELISA. HSPA1A: IL‐1β (A1 and A2), TNF‐α, (C1 and C2), IL‐10 (E1 and E2). HSPB1: IL‐1β (B1 and B2), TNF‐α, (D1 and D2), IL‐10 and (F1 and F2). Data are presented as mean ± SD, *n* = 3, and tested by two‐way ANOVA with Bonferroni's multiple comparison *post hoc*. Significant differences between either HSPA1A or HSPB1 and (either HSPA1A + CD14 or HSPB1 + CD14), (CD14 blocking peptide only) and control (HI‐RPMI) treatment are shown **P < 0.01 and ***P < 0.001.

**Fig. 9:**
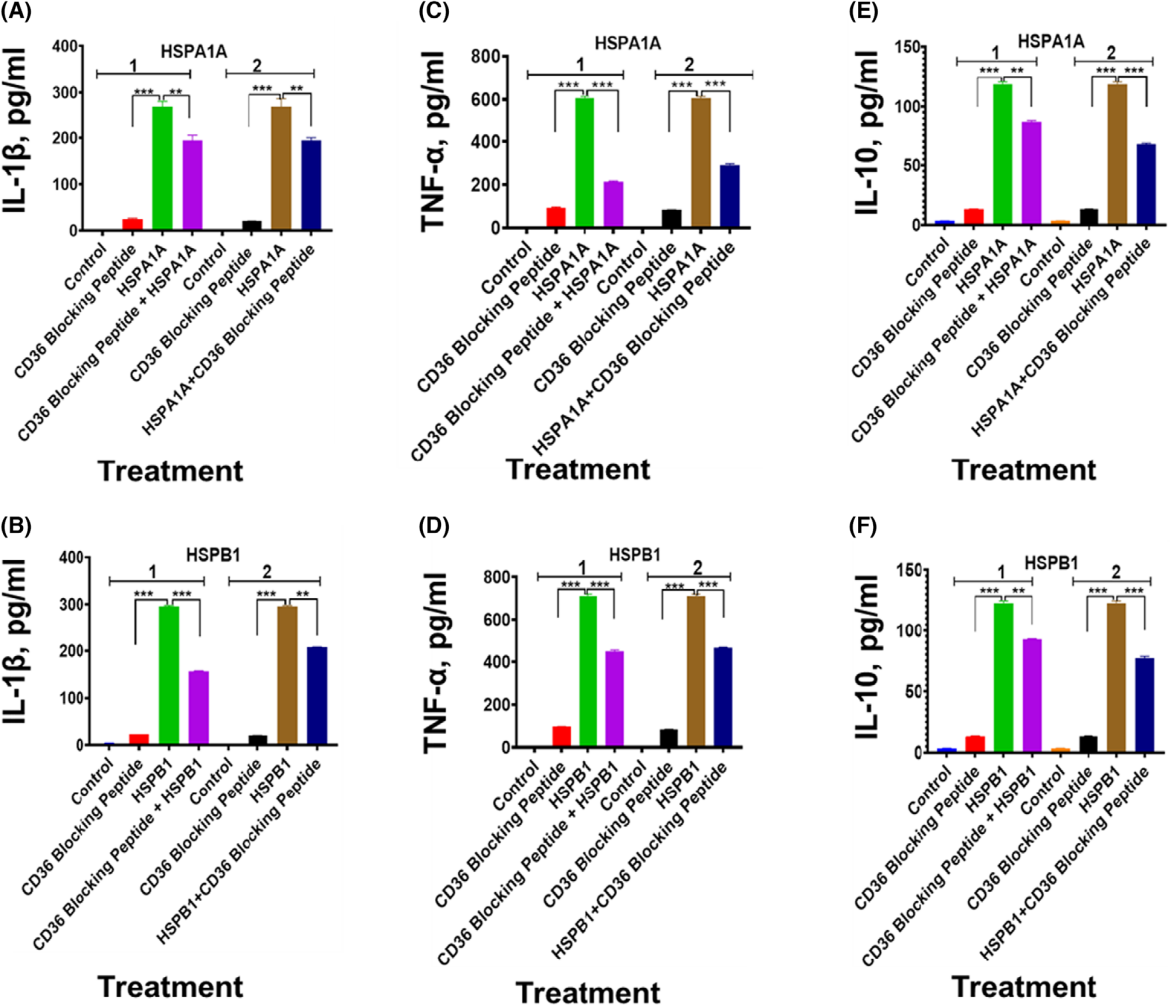
Effect of CD36 blocking peptide on either HSPA1A or HSPB1 induced IL‐1β, TNF‐α and IL‐10 secretion from differentiated U937 cells. Panels (A1 ‐F1) are results gained with differentiated U937 cells treated with 20 μg·mL^−1^ of CD36 blocking peptide alone and then incubated for 2 h followed by the addition of either 1000 ng·mL^−1^ of HSPA1A (A1, C1 and E1) or HSPB1 (B1, D1 and F1) and incubated for further 6 h. The cell suspension was then centrifuged, and the supernatant retrieved was measured for IL‐1β, TNF‐α and IL‐10 secretion into the media by ELISA. In addition, Panels (A2–F2) are results gained with 1000 ng·mL^−1^ of either HSPA1A (A2, C2 and E2) or HSPB1 (B2, D2 and F2) first preincubated with 20 μg·mL^−1^ of CD36 blocking peptide for 2 h, before applied to the differentiated U937 cells and incubated for 6 h. The cell suspension was then centrifuged, and the supernatant retrieved was measured for IL‐1β, TNF‐α and IL‐10 secretion by ELISA. HSPA1A: IL‐1β (A1 and A2), TNF‐α, (C1 and C2), IL‐10 (E1 and E2). HSPB1: IL‐1β (B1 and B2), TNF‐α, (D1 and D2), IL‐10 and (F1 and F2). Data are presented as mean ± SD, *n* = 3, and tested by two‐way ANOVA with Bonferroni's multiple comparison *post hoc*. Significant differences between either HSPA1A or HSPB1 and (either HSPA1A + CD36 or HSPB1 + CD36), (CD36 blocking peptide only) and control (HI‐RPMI) treatment are shown **P < 0.01 and ***P < 0.001.

**Fig. 10:**
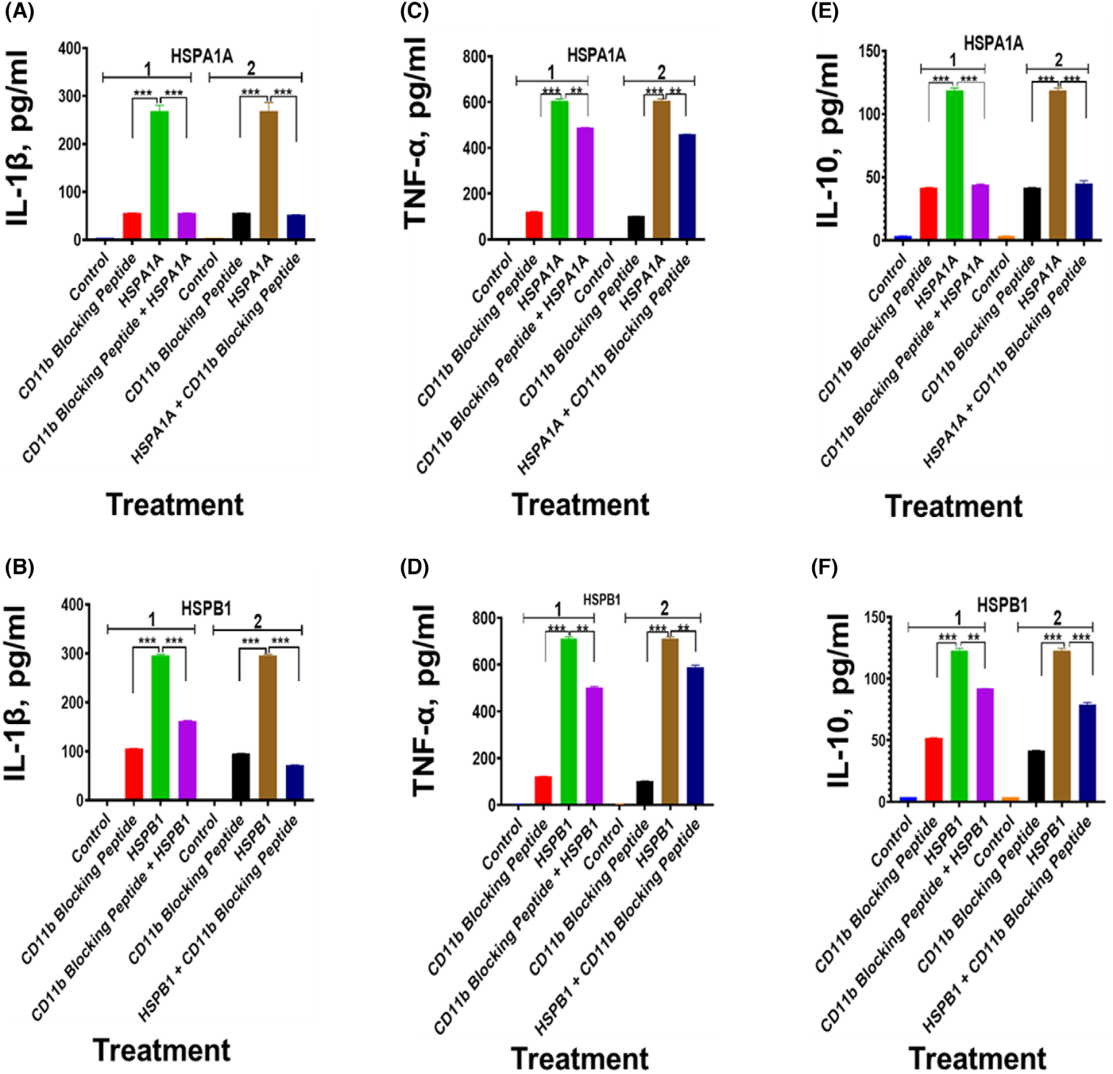
Effect of CD11b blocking peptide on either HSPA1A‐ or HSPB1‐induced IL‐1β, TNF‐α and IL‐10 secretion from differentiated U937 cells. Panels (A1–F1) are results gained with differentiated U937 cells treated with 20 μg·mL^−1^ of CD11b blocking peptide alone and then incubated for 2 h followed by the addition of either 1000 ng·mL^−1^ of HSPA1A (A1, C1 and E1) or HSPB1 (B1, D1 and F1) and incubated for further 6 h. The cell suspension was then centrifuged, and the supernatant retrieved was measured for IL‐1β, TNF‐α and IL‐10 secretion into the media by ELISA. In addition, Panels (A2–F2) are results gained with 1000 ng·mL^−1^ of either HSPA1A (A2, C2 and E2) or HSPB1 (B2, D2 and F2) first preincubated with 20 μg·mL^−1^ of CD11b blocking peptide for 2 h, before applied to the differentiated U937 cells and incubated for 6 h. The cell suspension was then centrifuged, and the supernatant retrieved was measured for IL‐1β, TNF‐α and IL‐10 secretion by ELISA. HSPA1A: IL‐1β (A1 and A2), TNF‐α, (C1 and C2) and IL‐10 (E1 and E2). HSPB1: IL‐1β (B1 and B2), TNF‐α, (D1 and D2), IL‐10 and (F1 and F2). Data are presented as mean ± SD, *n* = 3, and tested by two‐way ANOVA with Bonferroni's multiple comparison *post hoc*. Significant differences between either HSPA1A or HSPB1 and (either HSPA1A + CD11b or HSPB1 + CD11b), (CD11b blocking peptide only) and control (HI‐RPMI) treatment are shown **P < 0.01 and ***P < 0.001.

The preincubation of differentiated U937 cells with CD14 blocking peptide before either HSPA1A (Fig. 8A,C,E) or HSPB1 (Fig. 8B,D,F) was applied to the cells showed a significant (*P* < 0.001–*P* < 0.01) decrease in IL‐1β (Fig. 8A,B), TNF‐α (Fig. 8C,D) and IL‐10 (Fig. 8E,F) secretion, when compared to differentiated U937 cells treated with either HSPA1A or HSPB1 only (Fig. 8). The same treatment with CD36 blocking peptide showed a significant (*P* < 0.001–*P* < 0.01) decrease in IL‐1β (Fig. 9A,B), TNF‐α (Fig. 9C,D) and IL‐10 (Fig. 9E,F) secretion, when compared to differentiated U937 cells treated with either HSPA1A or HSPB1 only (Fig. 9). Similarly, the treatment with CD11b blocking peptide showed a significant (*P* < 0.001–*P* < 0.01) decrease in IL‐1β (Fig. 10A,B), TNF‐α (Fig. 10C,D) and IL‐10 (Fig. 10E,F) secretion, when compared to differentiated U937 cells treated with either HSPA1A or HSPB1 only (Fig. 10). Similar responses were also observed when either HSPA1A or HSPB1 was preincubated with either CD14, CD36 or CD11b blocking peptide before applied to differentiated U937 cells compared with HSPA1A or HSPB1 treatment alone (Figs 8, 9, 10).

## Discussion

In this study, using ELISA, extracellular HSPA1A and HSPB1 were shown to induce cytokine secretion in naïve and activated cells. A number of authors have reported cytokine responses to HSPs [28, 29, 42, 43]; however, this observation has been criticised by Gao and Tsan [44, 45] who found that, in their hands, contaminating LPS was responsible for this activity. Henderson *et al*. [46] reviewed the evidence for the cytokine‐stimulating activity of HSPs and were able to conclude that HSPs do have signalling activity. However, it is still important to demonstrate that any signalling activities are due to the HSP proteins and not to bacterial contaminants. In this current study, we have confirmed the signalling activity of HSP through the observations that both denaturing the HSP and incubation with specific HSP antibodies eliminate the signalling activity.

Cytokines are generally used as a biomarker that indicates an immune response. The immune response may be either predominantly pro‐ or anti‐inflammatory [47, 48]. Pro‐inflammatory cytokines, including IL‐1β and TNF‐α, are predominant during pro‐inflammatory processes, such as pathological pain [49]. Anti‐inflammatory cytokines such as IL‐10 are immunoregulatory molecules that control pro‐inflammatory immune responses [48]. Generally, in an acute immune response, TNF‐α and IL‐1β are initially secreted [50, 51] and their secretion activates secondary immune responses. IL‐10 is then secreted resulting in an initiation of resolution of the inflammation [48]. TNF‐α, IL‐1β and IL‐10 are therefore suitable cytokines to use as biomarkers of inflammatory responses [48]. U937 monocytic cells show typical human monocyte responses and is one of the most commonly used model for investigating monocyte immune function. PMA is commonly used in the study of U937 cell differentiation to macrophages [52, 53]. The primary cells (PBMCs) were also included in this study to determine that the effects observed in the U937 cell are reflective of *in vivo* responses.

Firstly, in this study, naïve U937 cells treated with different concentrations of either HSPA1A or HSPB1 did not secrete TNF‐α or IL‐1β while low levels of IL‐10 were secreted. Conversely, the same treatment using differentiated U937 cells resulted in significant secretion of TNF‐α, IL‐1β and IL‐10, consistent with previous data [42]. The level of IL‐10 secretion in differentiated U937 cells was significantly higher than that in naïve U937 cells. These results confirm the pro‐inflammatory response of differentiated monocytes when challenged with danger signals [52, 54, 55] as well as the ability of HSPA1A and HSPB1 to induce both pro‐ and anti‐inflammatory cytokines [42]. Therefore, the level of HSPA1A or HSPB1‐induced cytokines was dependent on the concentration of HSPs, and the type of cells involved, consistent with other studies [43, 56, 57]. The results obtained in this study showed that a cytokine secretion was induced by 10 ng·mL^−1^ HSP and was maximal in cells treated with 1000 ng·mL^−1^ of either HSPA1A or HSPB1. A time course of the responses showed that TNF‐α and IL‐1β are rapidly secreted in response to either HSPA1A or HSPB1 with IL‐10 secretion being slower—typical of the response of immune cells to danger signals [48, 50, 51].

It is also important to mention that cell responses to HSPs were not identical between differentiated U937 cells and PBMCs. For example, TNF‐α secretion was higher and IL‐10 secretion was lower in PBMCs. PBMCs comprise of several cell types, and this observation confirmed that the nature of a cell can play a role in the level of cytokine secretion, and this can be associated with the concentration of extracellular receptors present in the cell [53, 58].

The differentiated U937 cells in this study expressed CD14, CD36 and CD11b. HSP interaction with CD14, CD36 and CD11b was further investigated using blocking peptides. The results showed that both HSPA1A and HSPB1 were able to interact with extracellular CD14, CD36 and CD11b to induce IL‐1β, TNF‐α and IL‐10 production. CD14 has been reported as a co‐receptor of HSPA1A [28]. HSPA1A was shown to stimulate pro‐inflammatory cytokine production through two routes, one mediated by CD14 [28], and involving TLR2 and TLR4 [59]. HSPA1A has also been shown to interact with scavenger receptors LOX‐1, SREC‐1 and STABILIN‐1 [60]. These studies have all highlighted pro‐inflammatory responses, whereas in this current study, we also observed an anti‐inflammatory response through the production of IL‐10. *In vivo* studies with mice and humans have shown that the net response to HSPA1A (or inducible Hsp70 in mice) is anti‐inflammatory [61, 62]. In mice, Hsp70 inhibits inflammation by mediating degradation of the p65 subunit of NF‐kbeta [63] and reduces inflammation through iTregs [64]. As with HSPA1A, we observed that HSPB1 stimulated both pro‐inflammatory and anti‐inflammatory cytokine production. HSPB1 has also been shown to have anti‐inflammatory activity, through interaction with CD36 and SR‐A1 [65].

Both HSPA1A and HSPB1 seem to interact with numerous receptors—mostly scavenger receptors. LPS also interacts with several scavenger receptors—CD14 [33], SR‐A1 [34], CD36 [35, 36] and CD11b [37]. In the study presented here, using immunocytochemistry and flow cytometry, CD14, CD36 and CD11b were confirmed in the monocytic cells used (Figs 5, 6, 7). However, other receptors may also play a role in these cells. Furthermore, damage‐associated molecular patterns (DAMPs) and pathogen‐associated molecular patterns (PAMPs) interact with CD14, and their responses are mediated through TLR2 and/or TLR4—for example high mobility group box 1 (HMGB1) [66] and uric acid [67]. There is, therefore, significant promiscuity between these signalling DAMPs and their receptors. Additional experiments presented here showed that using blocking peptides for CD14, CD36 and CD11b induced lower secretion of IL‐1β, TNF‐α and IL‐10 when exposed to the HSPs compared with HSPs alone. This could be due to cell sensitization following blocking peptides and extracellular receptor complex formation. However, the results in this study showed that blocking peptides were not able to completely inhibit HSPA1A and HSPB1 induced IL‐1β, TNF‐α and IL‐10 secretion (Figs 8, 9, 10). These results would indicate either that the concentration of blocking peptides used was not sufficient to fully saturate their respective receptor or, that other as yet, unidentified receptors were also involved in this process. Furthermore, it cannot exclude that HSP induces cytokine secretion response may require the binding to multiple receptor types acting in a synergistic manner in order to illicit a full response.

Both HSPA1A and HSPB1 are present in serum and the extracellular environment, whether free or in exosomes, and concentrations alter during disease [8, 9, 10, 11, 12, 68], and they have potential to be used as indicators of disease progression [68, 69]. The promiscuous nature of HSPA1A and HSPB1 with extracellular receptors indicates the possibility of synergies between HSP and other danger signals such as HMGB1, and with bacterial PAMPs such as LPS. This promiscuity may allow HSPs, including HSPA1A and HSPB1, flexibility in responding to pathogens and other sterile threats, but also increases the potential for cell and tissue damage or autoimmunity if for any reason the anti‐inflammatory response is inhibited. Greater knowledge on the presence of other DAMPs and which receptors may be of importance in specific diseases and individuals and may improve the therapeutic potential for HSP modifiers. The results in this study have shown that HSPA1A and HSPB1 can activate both pro‐inflammatory and anti‐inflammatory cytokines secretion in naïve and differentiated U937 cells. We have also shown, in these cells, that CD14, CD36 and CD11b contribute to both HSPA1A‐ and HSPB1‐induced cytokine secretion. We have then shown that HSPA1A and HSPB1 can activate both pro‐inflammatory and anti‐inflammatory cytokines secretion from non‐transformed cells—PBMCs. PBMCs typically comprise 70–90% lymphocytes, 10–20% monocytes and 1–2% dendritic cells. We cannot say from these data which cells were responsible for this cytokine secretion; however, CD14 is expressed on activated monocytes and T cells [70]; CD36 is expressed on activated monocytes [71], and CD11b is expressed on monocytes [72]. Future studies should investigate the role of the individual cell populations in responding to HSPA1A and HSPB1. CD14 is linked to TLRs, and it would be interesting to determine whether TLRs also play a role in HSPA1A, and HSPB1‐induced cytokine production. The effect of blocking peptides in HSPs‐induced cytokine production can also be used to investigate whether they can regulate other effector pathways that involve NF‐kB and MAPKs.

## Conclusion

This study has confirmed that extracellular HSPA1A and HSPB1 can activate cytokine secretion and further demonstrated the possible impact of CD14, CD36 and CD11b as a major contribution to the activation level of IL‐1β, TNF‐α and IL‐10 secretion. It is, however, very likely that other receptors are contributing to this observation, and there is, therefore, a need to investigate other receptors in this respect. Investigating whether blocking peptides can inhibit other appropriate receptors. For example, determining whether CD14 blocking peptide inhibits cytokine production in response to TLR4 or TLR2 agonists could have strengthen HSPs interaction with the receptors. Also, it will be interesting to investigate HSPA1A and HSPB1 interactions with receptors using other methods such as surface plasma resonance (SPR), small interfering RNA (siRNA) or clustered regularly interspaced short palindromic (CRISPR), which could show the functional role of these receptors during HSP‐induced cytokine production in cells. These findings might also provide the rationale for an investigation into the use of exogenous HSP including HSPA1A and HSPB1 to alter the immune system for therapeutic purposes.

## Conflict of interest

The authors declare no conflict of interest.

### Peer review

The peer review history for this article is available at https://www.webofscience.com/api/gateway/wos/peer‐review/10.1002/2211‐5463.13695.

## Author contributions

EO and JHHW conceived the study and designed the experiments. JHHW and FM supervised the study. EO executed the experiments and analysed the data. EO, JHHW and FM interpreted the data and planned the publication. EO wrote the first draft of the manuscript and all authors edited and approved of the manuscript.

## Data Availability

The data that support the findings of this study are available from the corresponding author [ae2465@coventry.ac.uk and ogbodoemmanuel3@gmail.com] upon reasonable request.
